# Supplementary material for: Regulation of lymphoma in vitro by CLP36 through the PI3K/AKT/CREB signaling pathway
Source: PeerJ. 2024 Dec 24;12:e18693. doi: 10.7717/peerj.18693 (PMC11674146; doi:10.7717/peerj.18693)
Supplement: Supplemental Information 1 [file peerj-12-18693-s001.zip › please see all WB for original bands.pptx]

## Slide 1
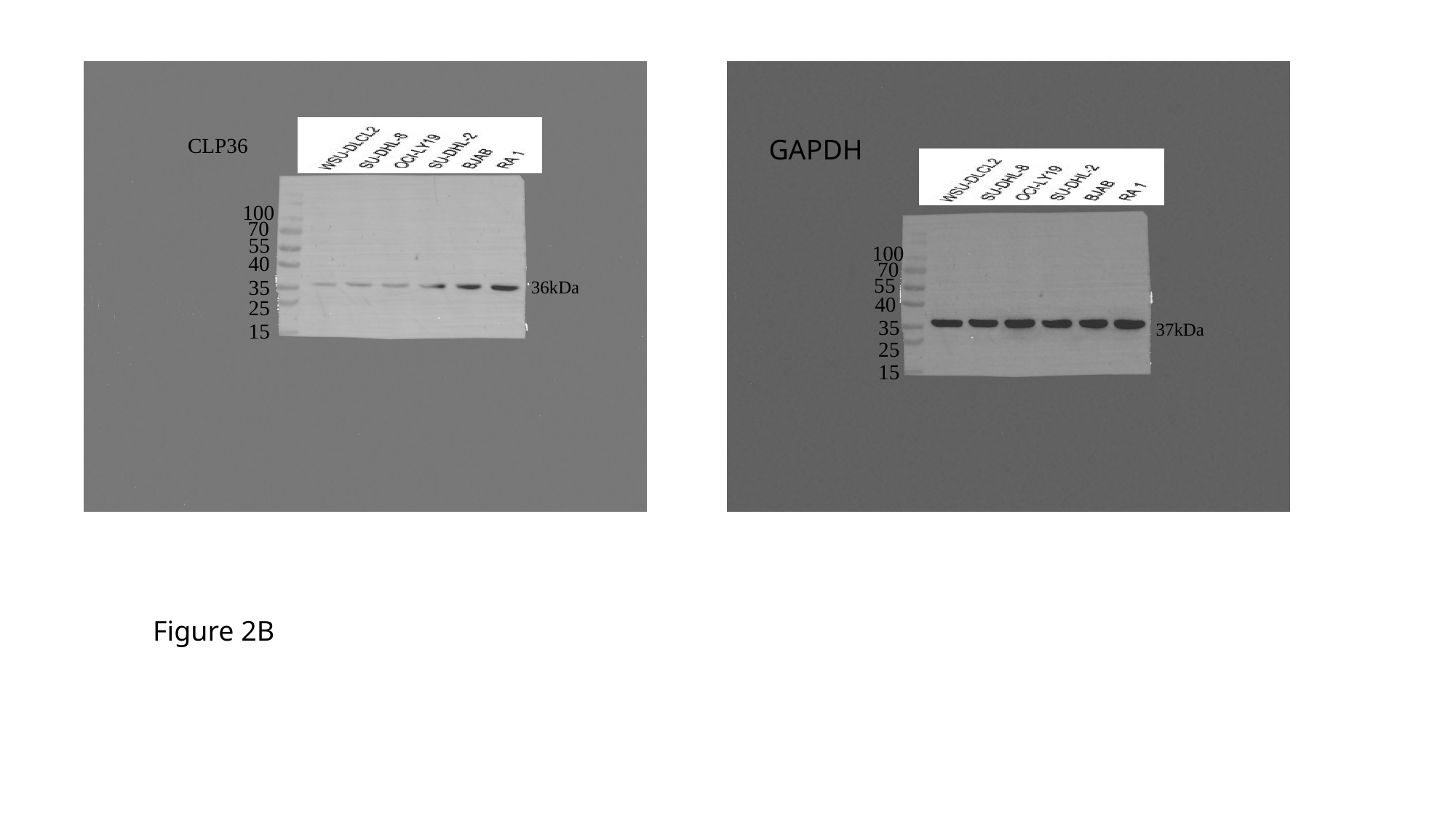

CLP36
GAPDH
100
70
55
100
40
70
55
35
36kDa
40
25
35
15
37kDa
25
15
Figure 2B

## Slide 2
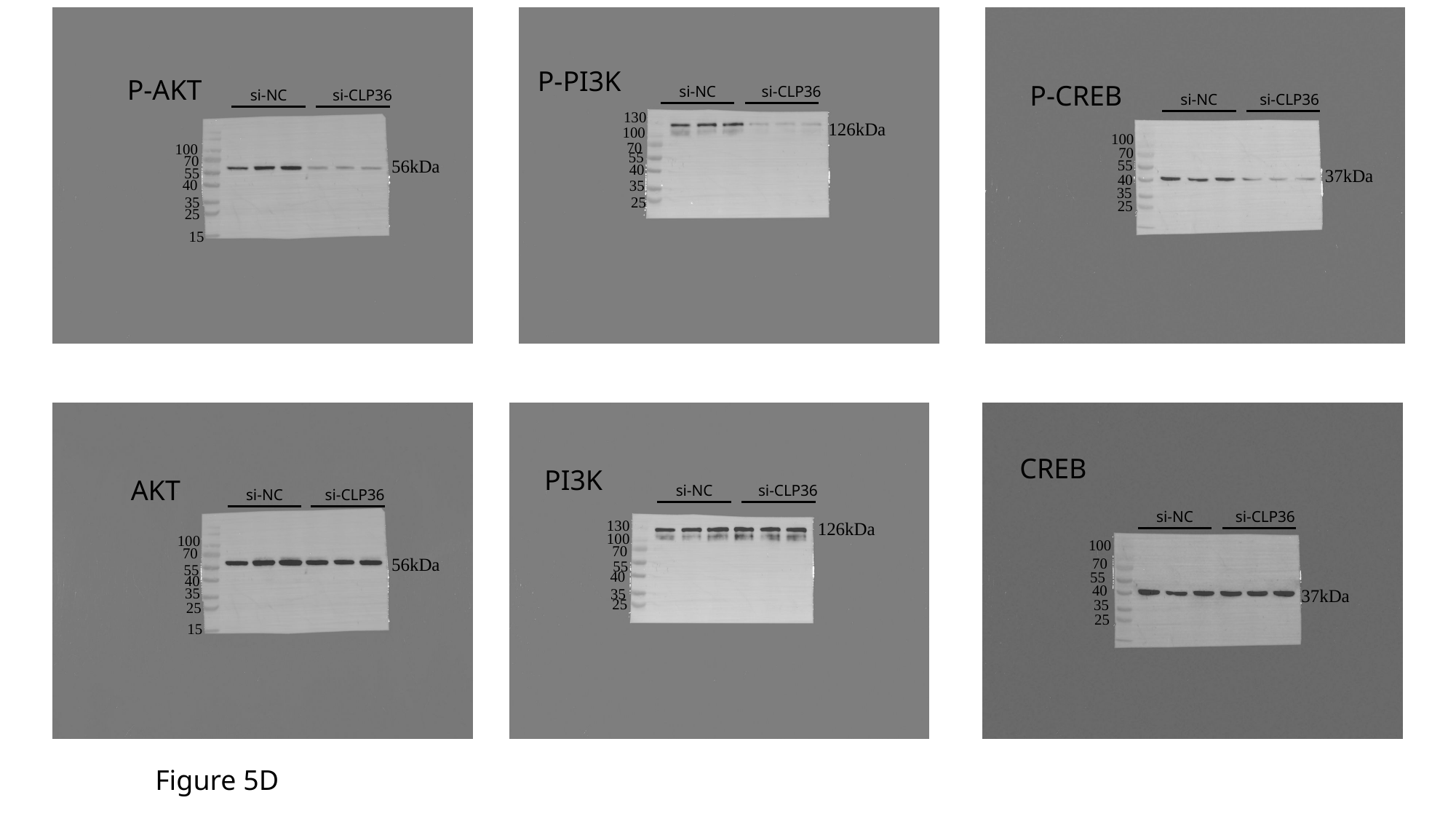

P-PI3K
P-AKT
P-CREB
si-NC
si-CLP36
si-NC
si-CLP36
si-NC
si-CLP36
130
126kDa
100
100
70
100
70
55
70
56kDa
55
40
55
37kDa
40
40
35
35
35
25
25
25
15
CREB
PI3K
AKT
si-NC
si-CLP36
si-NC
si-CLP36
si-NC
si-CLP36
130
126kDa
100
100
100
70
70
56kDa
70
55
55
40
55
40
40
35
35
37kDa
25
35
25
25
15
Figure 5D
